# Morphology-Engineered NiMo Alloy on Nickel Foam for Enhanced Hydrogen Evolution Reaction Performance

**DOI:** 10.3390/molecules30112396

**Published:** 2025-05-30

**Authors:** Yanhong Ding, Yong Cao, Zhichao Gao, Hanzhou Ding, Haifeng Xu, Bin Liu, Fusheng Liu, Yirong Zhu

**Affiliations:** School of Materials Science and Engineering, Hunan University of Technology, Zhuzhou 412007, China; dyh2004@126.com (Y.D.); qq04261998@163.com (Y.C.); gzc20201222@126.com (Z.G.); 18974956112@163.com (H.D.); 19174423169@163.com (H.X.); 15170898688@163.com (B.L.); liucsu@gmail.com (F.L.)

**Keywords:** NiMo alloy, water electrolysis, electrocatalysis, hydrogen evolution performance

## Abstract

A nanoflower-like nickel-molybdenum alloy was synthesized by hydrothermal in situ growth of NiMoO_4_ nanorod arrays on nickel foam (NF) followed by gas-phase re-reduction at 600 °C. The resulting structure has a uniform porosity and high specific surface area, which improves the availability of active sites and facilitates efficient electron and mass transport. SEM and XPS analyses confirm that the formed NiMoO_4_ nanorods are uniformly distributed, which leads to significant optimization of their electronic structure. The electrochemical measurements revealed that the sample exhibited excellent hydrogen evolution reaction (HER) performance, with an overpotential as low as 127 mV at 100 mA cm^−2^ and a Tafel slope of 124 mV dec^−1^. CV and EIS showed that the sample had the largest electrochemically active surface area (121.3 mF cm^−2^) among the samples treated at different temperatures, with the smallest charge transfer resistance. In addition, the catalyst maintained high stability after 45 h of continuous operation. These results highlight the potential of NiMo/NF as a highly efficient and durable HER catalyst to help advance hydrogen energy technology.

## 1. Introduction

The escalating severity of global climate challenges, coupled with the progressive depletion of fossil fuel reserves, has rendered energy transition an urgent global imperative. This critical juncture positions clean energy technologies as a pivotal strategy for concurrently addressing climate change mitigation and sustainable energy security [1,2,3,4,5,6]. Among various clean energy technologies, water electrolysis is regarded as a promising approach for producing green hydrogen, as it enables the clean conversion of water into hydrogen and oxygen using electricity, yielding a high energy-density, emission-free fuel [7,8]. Coupling water electrolysis with renewable energy sources such as wind and solar mitigates their intermittency, reduces carbon emissions, and enhances energy efficiency and sustainability [9,10,11,12]. As a result, water electrolysis for hydrogen production is gaining increasing attention as a key component of future clean energy systems [13].

Although water electrolysis offers high theoretical efficiency, its practical performance is hindered by the sluggish kinetics of the hydrogen evolution reaction (HER), which demands a high overpotential to overcome energy barriers, resulting in elevated energy consumption and production costs [14,15,16,17]. Precious metal-based electrocatalysts, such as platinum and its alloys, are regarded as the most effective HER catalysts due to their superior activity and low overpotential. However, their high cost and limited availability present major obstacles to large-scale deployment [18,19,20]. Therefore, the development of non-precious metal-based HER catalysts with high catalytic performance and low cost has become the key to solving this problem. In recent years, transition metals have become a hot spot in electrocatalyst research due to their abundant resources, low cost and excellent electrochemical performance, among which nickel–molybdenum (NiMo) alloys have attracted much attention due to their unique electronic structure and excellent HER catalytic performance [21,22,23]. It has been shown that the catalytic activity of NiMo alloys can be significantly enhanced by modulating the component ratio, optimizing its surface structure and compounding with other functional materials [24,25]. However, the traditional NiMo alloy catalysts still face the problems of insufficient exposure of active sites, limited mass transfer, and poor electrical conductivity in practical applications, and these bottlenecks seriously limit their industrial promotion [26,27,28,29]. Consequently, optimizing the structure and performance of NiMo alloys while preserving their intrinsic advantages has become a key focus of current research [30,31,32,33,34,35].

To address the aforementioned limitations, this study presents a structural engineering strategy for NiMo alloy catalysts supported on nickel foam, aiming to enhance HER performance through material optimization. NiMo alloys were synthesized on a high-surface-area, three-dimensional porous nickel foam via a combined hydrothermal and gas-phase reduction method. The nickel foam not only ensured excellent electrical conductivity but also increased the exposure of active sites, thereby improving catalytic activity. Additionally, the NiMo alloy’s electronic structure was tailored by controlling crystal growth orientation and surface morphology, effectively reducing the overpotential for HER. The gas-phase reduction further promoted alloy uniformity and stability, contributing to long-term electrochemical durability. The catalyst’s performance was systematically evaluated, demonstrating its potential as a cost-effective and efficient material for hydrogen production via water electrolysis.

## 2. Results and Discussion

### 2.1. Structural Topography Analysis

**Figure 1:**
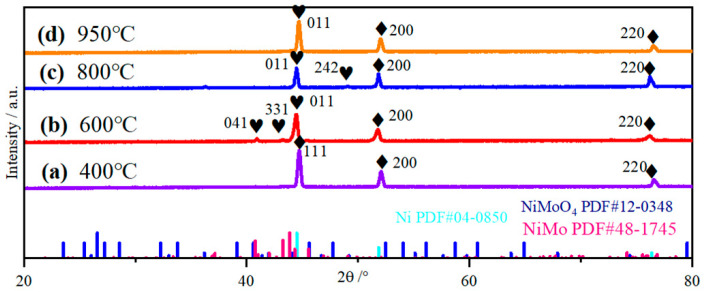
XRD images of NiMo/NF at different reduction temperatures.

Figure 1 illustrates the X-ray diffraction (XRD) spectra of four NiMo/NF electrode samples prepared at different reduction temperatures (the ‘heart’ and ‘diamond’ symbols in the figure represent Ni and NiMo, respectively). It can be observed that diffraction peaks corresponding to the (111), (200) and (220) crystal planes appear at 44.50°, 51.85°, and 76.38°, respectively, which are in agreement with the standard data for NF (PDF#04-0850). Under the reduction conditions at 600 °C, 800 °C, and 950 °C (Figure 1b–d), the diffraction peaks of the (200) and (220) crystal planes were shifted to the left, indicating an increase in the crystal plane spacing, which may be due to the doping of Mo ions, which have a larger radius than that of Ni ions. In addition, diffraction peaks corresponding to the (011), (041), (331) and (242) crystal planes appeared at 44.34°, 41°, 43°, and 49.18°, respectively, which were consistent with the standard data for NiMo alloys (PDF#48-1745) [36]. In Figure 1a, the diffraction peaks of NiMo alloy were not observed, which may be due to the low reduction temperature (400 °C), which did not allow a sufficient reduction reaction to take place. The differences in atomic radii and electronegativity of Ni and Mo resulted in lattice distortion, which led to the formation of diverse structures on some of the crystalline surfaces, which manifested as new NiMo alloy diffraction peaks, such as (041) and (331) crystalline peaks (Figure 1b) and (242) crystalline peaks (Figure 1c). The relatively weak intensity and broadening of these diffraction peaks [37] indicate the presence of defects in the crystal structure, leading to an increase in surface energy [37]. These structural changes contribute to the enhancement of the hydrogen evolution reaction (HER) activity, which significantly increases the hydrogen precipitation rate. When the reduction temperature was raised to 950 °C, the diffusion rates of Ni and Mo ions increased, facilitating the formation of Ni-Mo alloys. Consequently, the intensity of the diffraction peaks corresponding to the (011) crystal plane of NiMo was significantly enhanced (see Figure 1d), indicating an improvement in the integrity of the crystal structure [38,39,40]. In addition, by comparing with the standard data of Ni (PDF#48-1745), no obvious NiMoO_4_ diffraction peaks were found, which indicates that NiMoO_4_ has amorphous structural properties.

### 2.2. SEM Analysis

**Figure 2:**
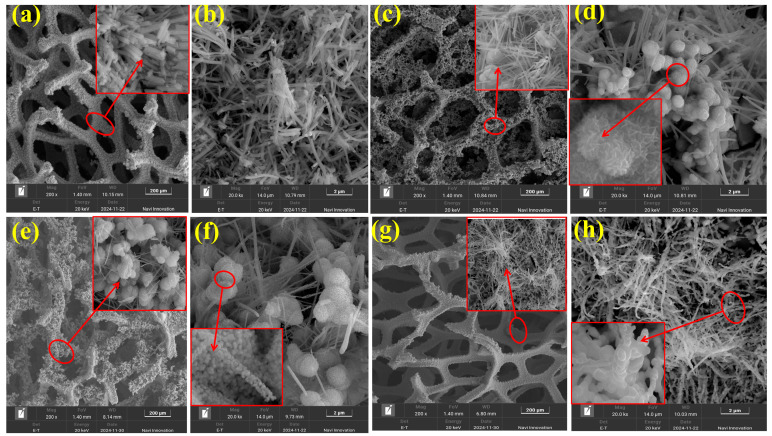
(**a**,**b**) SEM images of NiMo/NF at 400 °C; (**c**,**d**) SEM images of NiMo/NF at 600 °C; (**e**,**f**) SEM images of NiMo/NF at 800 °C; (**g**,**h**) SEM images of NiMo/NF at 950 °C.

The microscopic morphological features of the electrodes were systematically investigated through scanning electron microscopy (SEM) characterization, as presented in Figure 2. Energy-dispersive spectroscopic analysis confirmed the substantial deposition of active materials onto the three-dimensional nickel foam substrate with an interconnected porous architecture. High-magnification images (inset in Figure 2a) revealed the presence of rod-shaped nanostructures with amorphous characteristics, which were subsequently identified as NiMoO_4_ through comparative XRD pattern analysis (Figure 1). Notably, these nanorods demonstrated heterogeneous spatial distribution (Figure 2b) with longitudinal dimensions varying between 2 and 8 μm. Thermal reduction treatment induced significant morphological evolution, where the elevated temperatures facilitated the enhancement of the diffusion kinetics of Ni and Mo species. This phenomenon promoted the heterogeneous nucleation processes, ultimately resulting in the surface decoration of nanorods with spherical NiMo alloy particulates (inset in Figure 2c). The formation of metallic NiMo phases was further corroborated by the emergence of characteristic (111) and (200) diffraction peaks in corresponding XRD patterns (Figure 1), confirming the successful phase transformation under reductive thermal conditions [41].These spherical particles have a diameter of about 1 µm and a uniform particle size distribution (Figure 2d). From the inset of Figure 2d, it can be clearly observed that these spherical particles exhibit a flower-spherical shape, whose surface is not smooth but consists of a uniform nanosheet structure. This flower-spherical structure not only significantly increases the specific surface area, but also exposes more catalytically active sites, which in turn shortens the diffusion paths of electrons and ions and reduces the transport resistance, thus accelerating the rate of the hydrogen evolution reaction (HER) and enhancing the electrocatalytic performance. Moreover, the flower-spherical structures synthesized at a high temperature of 600 °C show significant morphological advantages and excellent performance, which is mainly attributed to the following key factors: firstly, the high-temperature environment provides sufficient energy for the self-assembly process of the nanosheets, which promotes the orderly arrangement and directional growth of the nanosheets, resulting in the formation of a regular flower-spherical structure. This unique morphological feature not only endows the material with rich pore structure, but also significantly increases the specific surface area, providing more active sites for electrochemical reactions. Secondly, the high-temperature treatment helps to improve the crystallinity of the material and enhance its structural stability. In addition, the multilevel pore network of the flower-spherical structure facilitates the penetration of the electrolyte and the diffusion of the reaction products, further enhancing the electrochemical performance of the material. The synergistic effect of these structural advantages enabled the samples synthesized at 600 °C to exhibit significantly better catalytic activity in the electrochemical hydrogenolysis reaction (HER) than those prepared at other temperature conditions. The synthesis temperature of 600 °C induces three critical enhancements in material properties: (1) The hierarchical structure with abundant nanorod arrays creates substantial adsorption sites for reactive species (H_2_O, H^+^, OH^−^, etc.), significantly improving interfacial contact efficiency while establishing a stable microstructural framework. This ordered architecture not only reinforces mechanical stability but also establishes favorable preconditions for catalytic processes. (2) Elevated crystallinity accompanied by reduced lattice defects facilitates efficient charge carrier migration through two mechanisms: the minimized crystal imperfections reduce the probability of electron scattering, while the intimate interrod contact within the spherical-flower morphology effectively diminishes interfacial charge-transfer resistance, thereby optimizing electrochemical reaction kinetics. (3) Thermally optimized elemental distribution enables electronic synergy between Ni and Mo components—the d-electron-enriched Ni sites preferentially adsorb and reduce H^+^ species, while high-valent Mo centers promote O_2_ evolution through oxidative pathways. This bimetal synergy, combined with their homogeneous dispersion in the spherical-flower matrix, generates maximized interfacial active sites that significantly boost hydrogen evolution reaction (HER) efficiency. Collectively, the structural advantages of the temperature of 600 °C, including enhanced specific surface area, optimized electronic configuration, and improved charge transfer capability, synergistically contribute to the superior electrocatalytic performance, positioning these materials as promising candidates for high-efficiency hydrogen evolution applications [42,43,44,45]. Elevating the reduction temperature beyond optimal levels induced two sequential morphological transitions: (1) At intermediate temperatures, the density of spherical particles increased with concomitant size polydispersity (inset in Figure 2e), while the lamellar architectures progressively evolved into smooth-surfaced spherical superstructures composed of subunit assemblies (inset in Figure 2f), which is attributed to thermally accelerated Ni-Mo alloying dynamics. (2) Upon reaching the critical threshold of 950 °C, complete structural reorganization occurred, marked by nanorod dissolution (inset in Figure 2g) and replacement of spherical morphologies with linear configurations featuring multi-nodal strand assemblies (inset in Figure 2h). This terminal transition confirms exhaustive alloying of the rod-like NiMoO_4_ precursor and full oxygen reduction, which is consistent with the emergence of dominant NiMo intermetallic phases in XRD analysis (Figure 2d) [46]. In summary, SEM analyses showed that the specimens with lamellar flower ball structure exhibited better electrocatalytic properties at the reduction temperature of 600 °C.

**Figure 3:**
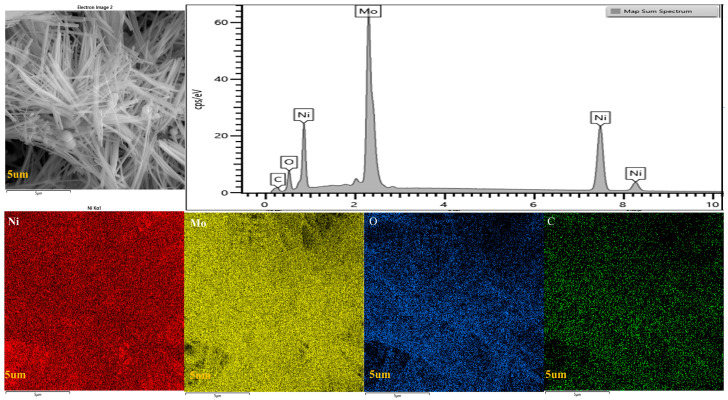
EDS scan of a NiMo-600 °C/NF sample.

To systematically investigate the surface elemental distribution of NiMo-600 °C/NF composites, energy-dispersive spectroscopy (EDS) mapping was conducted within a representative 5 µm microregion (Figure 3). The EDS spectra exhibit prominent characteristic peaks of Ni and Mo, confirming their dominance in the surface composition. Corresponding elemental mapping reveals spatially consistent distributions of both metallic components across the nickel foam (NF) substrate, demonstrating successful homogeneous loading of NiMo species onto the three-dimensional NF framework. This uniform dispersion is further evidenced by high-intensity superposition in the composite elemental mapping, indicative of a well-controlled synthesis process. Quantitative analysis of the atomic ratio highlights the predominant contribution of Ni and Mo, aligning with the designed stoichiometry of the NiMo alloy. The observed microstructural homogeneity not only validates the structural integrity of the NiMo/NF hybrid system but also suggests long-range ordering at the atomic level, a critical factor for stabilizing active sites during the electrocatalytic processes. These findings collectively establish a direct correlation between the synthesis-controlled elemental distribution and the enhanced functional performance of the composite material.

Combining the application of scanning electron microscopy (SEM) and EDS analysis, the relationship between the microstructure and the electrocatalytic performance of NiMo/NF materials can be more clearly understood. The systematic analysis of the elemental distribution can reveal the catalytic mechanism of NiMo composites, which provides an important theoretical basis and practical guidance for their potential practical application in electrocatalytic reactions. Further studies can focus on optimizing the loading and distribution of NiMo to enhance its catalytic activity and stability and to promote the wide application of this material in the field of energy conversion and storage.

### 2.3. XPS Analysis

**Figure 4:**
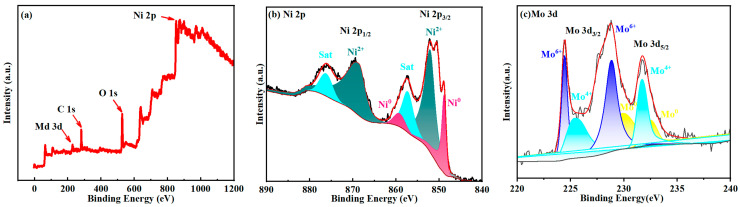
XPS spectra of NiMo-600 °C/NF sample: (**a**) full spectrum, (**b**) Ni 2p; (**c**) Mo 3d.

The valence states of the elements Ni and Mo in NiMo/NF and Ni/NF were analyzed by X-ray photoelectron spectroscopy (XPS). The full XPS spectra of NiMo/NF and Ni/NF (Figure 4a) show that the elemental compositions of NiMo/NF are Ni and Mo, whereas that of Ni/NF is Ni. The Ni 2p spectra of NiMo/NF are illustrated in Figure 4b. The peaks with binding energies of 870.2 eV and 852.6 eV are attributed to Ni^2+^, while satellite peaks are also observed at 876.4 eV and 857.4 eV. The presence of Ni^2+^ is attributed to the formation of oxides on the surface of NiMo/NF after exposure to air, a phenomenon that has been reported in previous studies. Ni^2+^ is able to accelerate the electron transfer to water molecules and promote the decomposition of water molecules, thus enhancing the HER performance. In addition, the peaks at 859.5 eV and 849 eV correspond to Ni^0^, indicating that NiMoO_4_ was successfully reduced during the annealing process. For the Ni 2p spectrum of Ni/NF (Figure 4b), the two main peaks with binding energies of 869.4 eV and 851.9 eV are likewise attributed to Ni^2+^, which is related to the formation of oxides from Ni/NF in air. Figure 4c presents the results of Mo 3d X-ray photoelectron spectroscopy (XPS) analysis conducted on NiMo/NF. The characteristic peaks observed at binding energies of 232.6 eV and 230 eV align with the metallic state of Mo^0^ (zero-valent molybdenum), providing further evidence that the NiMoO_4_ precursor was successfully reduced to a NiMo alloy during the annealing process. Additionally, four distinct peaks corresponding to Mo^4+^ (231.7 eV and 225.5 eV) and Mo^6+^ (228.8 eV and 224.5 eV) were identified in the spectra. The presence of these higher valence molybdenum species (Mo^4+^ and Mo^6+^) can be attributed to the formation of oxide layers on the surfaces of nickel–molybdenum alloys upon exposure to air. Importantly, the electron redistribution between nickel and molybdenum significantly optimizes the electronic structure of the material, which in turn enhances its catalytic activity. Specifically, the synergistic effect of Ni and Mo in NiMo alloys not only stabilizes reaction intermediates (such as H*, O*, etc.) but also promotes catalytic reaction kinetics by lowering the energy barriers associated with the reactions. In summary, the NiMo alloy produced through the annealing reduction method exhibits an optimized valence distribution and electronic structure, effectively enhancing catalytic reactions. This provides a crucial structural basis for its application in electrochemical hydrogenation reactions (HERs) and other fields [47,48].

### 2.4. Electrochemical Performance Analysis

**Figure 5:**
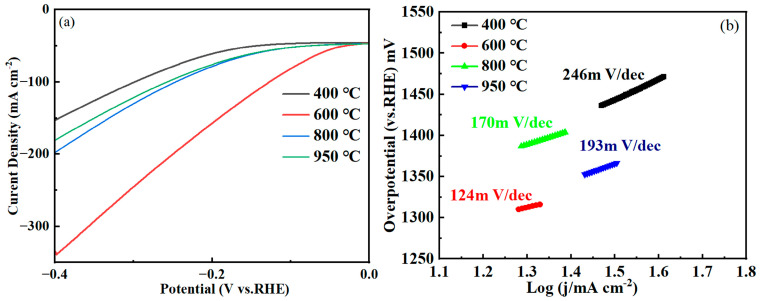
(**a**) Polarization curves of nickel–molybdenum alloy catalysts at different reduction temperatures; (**b**) Tafel plot.

By comparative analysis of the polarization curves of NiMo alloy catalysts prepared at different calcined reduction temperatures (Figure 5, with a scan rate of 5 mV/s), it can be clearly observed that the curves in Figure 5a indicate that the activity of the catalysts calcined and reduced at 600 °C is significantly better than that of the samples under other temperature conditions. Specifically, the 600 °C-treated Ni-Mo alloy catalyst exhibited excellent hydrogen evolution reaction (HER) performance with an overpotential η_100_ only 127 mV [49,50,51,52], which was significantly lower than that of the catalysts prepared under the other temperature conditions, suggesting that 600 °C is the optimal temperature for the preparation of high-performance Ni-Mo alloy catalysts. Figure 5b demonstrates the Tafel slope data of catalysts at different calcined reduction temperatures. Among them, the lowest Tafel slope was observed for NiMo 600 °C, followed by NiMo 800 °C, NiMo 950 °C and NiMo 400 °C in that order. This trend clearly indicates that 600 °C is the optimum temperature for the calcined reduction of the catalyst. At this temperature, the Tafel slope of the NiMo catalyst was 124 mV dec^−1^ [53,54]. The diminished Tafel slope demonstrates accelerated hydrogen evolution reaction (HER) kinetics through reduced activation energy barriers, manifesting as lower overpotential requirements at equivalent current densities that synergistically enhance interfacial charge transfer efficiency and current density escalation rates, thereby conclusively validating the superior electrochemical performance of 600 °C-synthesized NiMo/NF catalysts in facilitating energy-efficient hydrogen production. Through this optimized calcined–reduction protocol, the critical structure–activity correlation was established, providing mechanistic insights into Ni-Mo synergism while offering practical design principles for developing scalable electrocatalysts toward industrial hydrogen generation systems.

**Figure 6:**
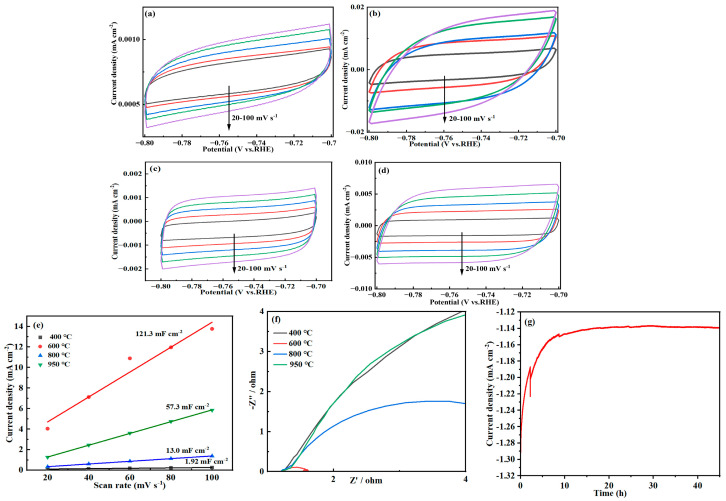
Cyclic voltammograms of nickel–molybdenum alloys in a 1.0 M NaOH solution at different scan rates in the non-Faradaic region under different calcination–reduction temperatures: (**a**) 400 °C, (**b**) 600 °C, (**c**) 800 °C, and (**d**) 950 °C. The scan range is from −0.8 V to −0.7 V vs. RHE, where the scan rates range from 20 mV/s to 100 mV/s, and the temperature is 25 °C. (**e**) The curve of the electrochemically active surface area of nickel–molybdenum alloy catalysts under different calcination–reduction temperatures. (**f**) Electrochemical impedance spectra of nickel–molybdenum alloy catalysts under different calcination–reduction temperatures. (**g**) Stability diagram of the nickel–molybdenum alloy catalyst at 600 °C measured by chronopotentiometry (CP).

In order to investigate the effect of different calcination temperatures on the catalytic hydrogen precipitation performance of Ni-Mo alloys, in this study, cyclic voltammetry (CV) was used to perform multi-scan rate tests (20–100 mV/s) in the non-Faraday region (−0.8 ~ −0.7 V vs. RHE), and the electrochemically active surface area (ECSA) of the catalysts was evaluated by calculating the electrochemical double layer capacitance (C_dl_). According to the equation C_dl_ = 1/2 Slope, the C_dl_ value was calculated using the slope of the CV curve. Figure 6a–d demonstrate the CV curves of NiMo alloy at 400 °C, 600 °C, 800 °C and 950 °C calcination temperatures, which shows that the oxidation peaks and reduction peaks are symmetrically distributed, and the peak heights are consistent with the peak spacing, which indicates that the redox reaction is easily reversible. Further analysis showed that the NiMo alloy catalyst reduced by calcination at 600 °C exhibited the highest ECSA (121.3 mF/cm^−2^, Figure 6e), which was significantly better than the samples at other temperature conditions [55]. This is due to the fact that in NiMo alloys, the electron density distributions of Ni and Mo are realigned. The difference between the higher electron density of Ni and the lower electron density of Mo promotes the transfer of electrons from Ni to Mo. This causes the 3d electron orbitals of Ni to interact with the 4d electron orbitals of Mo, resulting in a redistribution of the electron density. The addition of Ni modulates the electron density of Mo and lowers the d-band center of Mo, thus optimizing the hydrogen adsorption free energy (∆GH). Moreover, in NiMo alloys, there is a charge transfer phenomenon between Ni and Mo. Ni tends to provide electrons while Mo tends to accept electrons, and this charge transfer enhances the catalytic activity of the alloys. The electron density of Ni decreases and the electron density of Mo increases due to its electron transfer. This redistribution of electron density modulates the local electronic environment at the catalytically active sites. For the HER, the electron density of the catalytic active site needs to be moderate: too high an electron density may lead to too strong adsorption of hydrogen, making it difficult to desorb, while too low an electron density may lead to too weak adsorption of hydrogen, making it difficult to form the reaction intermediate. The electron transfer of Ni and Mo brings the electron density to an equilibrium state, thus optimizing the HER. This resulted in a significant increase in the density of active sites on the surface of the catalyst after treatment at 600 °C, and its multi-interfacial structure provided abundant catalytically active sites for the hydrogen evolution reaction (HER), and consequently enhanced the catalytic performance substantially. The electrochemical impedance spectroscopy (EIS) test results (Figure 6f) showed that the nickel–molybdenum alloy (NiMo) catalyst treated at 600 °C exhibited the smallest charge transfer resistance (R_ct_) [56,57]. In the Nyquist plot, the arc radius of the EIS spectral line is directly correlated with R_ct_, while the NiMo catalyst treated at 600 °C has the smallest arc radius of the EIS spectral line, indicating that its R_ct_ value is significantly lower than that of the samples treated at other temperatures. This result confirms that the catalyst treated at 600 °C has the least charge transfer resistance at the electrochemical interface and a more efficient electron transfer path. This excellent charge transfer property is mainly attributed to the following mechanisms: firstly, during the 600 °C treatment, the electron density of the 3d orbitals of Ni decreases while the number of holes increases. This change in electronic structure makes it easier for the Ni sites to adsorb H^+^ (protons), thus facilitating the Volmer step (H^+^ + e^−^ → H*) of the reduction of H^+^ to H* (adsorbed hydrogen atoms). Secondly, NiMo alloys form a more homogeneous crystal structure and tighter metal–metal bonding under high-temperature treatment, which significantly reduces the electron transport resistance within the material. In addition, the surface defects (e.g., oxygen vacancies) induced by the high-temperature treatment can act as ‘highways’ for electron transport, further accelerating the charge transfer process. At the same time, Mo sites are more prone to promote H desorption and H_2_; formation due to the increase in electron density and 4d-orbital electron density, thus optimizing the Tafel step (2H → H_2_) or Heyrovsky step (H* + H^+^ + e^−^ → H_2_) [58,59]. Therefore, the NiMo catalysts treated at 600 °C significantly enhanced the kinetic performance of the key steps of the HER (hydrogen adsorption and hydrogen desorption) by synergistically optimizing the electronic structures and surface defects of the Ni and Mo sites, providing an important theoretical basis for the design of high-efficiency non-precious metal catalysts. Since Ni has a stronger adsorption capacity for hydrogen but a weaker desorption capacity for Mo, the synergistic effect of Ni and Mo can regulate the free energy of hydrogen adsorption (∆GH*) to be close to the thermodynamic optimum (∆GH* ≈ 0), thus improving the reaction efficiency. The synergistic effect of nickel and molybdenum also improves the stability of the reaction intermediate (H), which further accelerates the reaction and provides efficient kinetic support for HER. During the long-term stability test, the NiMo catalyst treated at 600 °C demonstrated remarkable stability. As illustrated in Figure 6g, after 45 h of continuous operation using the constant current method at a high current density of 100 mA/cm^2^, the performance parameters showed no significant decline. This indicates that the structure of the active sites remained stable and no deactivation occurred [60]. These findings robustly confirm that the Ni-Mo alloy catalyst produced through calcination at 600 °C exhibits a combination of high activity, excellent stability, and efficient charge transfer capabilities. As such, it represents an ideal catalytic material for hydrogen evolution reactions (HERs) and serves as a crucial reference for advancing aquatic energy technologies.

## 3. Materials and Methods

**Figure 7:**
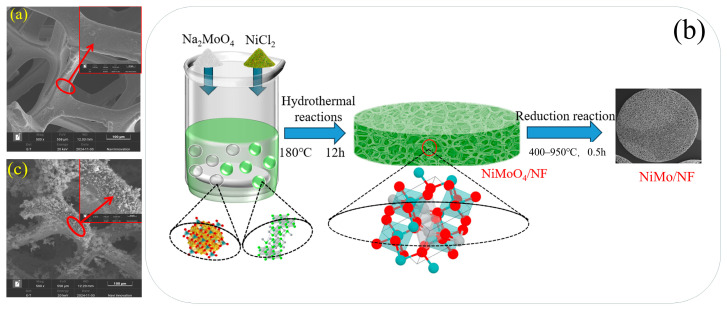
Preparation process and SEM images of NiMo/NF: (**a**) SEM image of NF; (**b**) process flow chart for the preparation of NiMo/NF; (**c**) SEM image of NiMoO_4_/NF.

In this study, NiMo/NF materials were successfully synthesized using nickel foam (NF) as a substrate by weighing 0.475 g of nickel chloride (NiCl_2_) and 0.484 g of sodium molybdate (Na_2_MoO_4_) using the hydrothermal method and gas phase reduction method. Its preparation process and micro-morphology were analyzed by SEM characterization (shown in Figure 7). Figure 7a demonstrates the porous network structure of NF with a smooth skeleton surface, which provides an ideal support platform for the subsequent growth of nanomaterials. The NiMoO_4_/NF precursor was synthesized by the hydrothermal method according to the chemical reaction equation Na_2_MoO_4_ + NiCl_2_ → NiMoO_4_ + 2NaCl (Figure 7b). The SEM image (Figure 7c) shows that the NF backbone is uniformly grown with a large number of NiMoO_4_ nanorods, indicating that the hydrothermal method has significant advantages in controlling the morphology of nanostructures. Subsequently, four NiMo/NF materials at different temperatures were prepared by gas-phase reduction reaction at 400 °C, 600 °C, 800 °C and 950 °C, according to the reaction equation NiMoO_4_ + 4H_2_ → NiMo + 4H_2_O, respectively. (Chemicals were purchased from McLean’s Reagent and NF was purchased from China Xing Zhenghong Technology Enterprise Store).

Nickel foam was selected as an ideal substrate due to its excellent electrical conductivity and high specific surface area. Nickel chloride and sodium molybdate served as metal precursors, offering the potential for high catalytic efficiency. Uniformly distributed NiMoO_4_ nanorods were synthesized under mild conditions via a hydrothermal method, followed by gas-phase reduction at various temperatures to form metallic NiMo. This combined approach ensures both the controllability and reproducibility of the synthesis process, providing a practical and scalable strategy for fabricating NiMo/NF materials with promising catalytic applications.

For the electrochemical test, a three-electrode system was tested using an electro- chemical workstation (Model CHI-760E Shanghai Chenhua Instrument Co., Ltd. is from Shanghai, China.). The three-electrode system consists of four catalyst samples (working electrode), a platinum sheet (counter electrode), and a Hg/HgO electrode (reference electrode). As for the washing of nickel foam, the cut nickel foam (1 cm × 1 cm) was washed thoroughly with acetone, 3 mol/L hydrochloric acid, anhydrous ethanol, and deionized water. The electrolyte solution is usually 1 mol/L NaOH. Test conditions were as follows: the electrocatalytic hydrogen polarization curve (scan speed of 5 mV/s) and impedance were tested using 1 mol/L NaOH electrolytes, and the electrode stability was tested using the constant current method.

## 4. Conclusions

In this study, NiMoO_4_ nanofiber precursors were successfully synthesized on nickel foam (NF) via a hydrothermal method, followed by gas-phase reduction to obtain NiMo alloys. This controllable and straightforward synthesis significantly enhances the electrochemical performance of the resulting electrodes. The synergistic interaction between Ni and Mo, combined with the 3D porous nanostructure, increases the electrochemically active surface area (ECSA) and active site density, thereby improving HER kinetics. The porous structure also enlarges the contact area, reducing contact resistance and ensuring uniform current distribution, which minimizes localized heating and enhances overall conductivity. In alkaline media, the NiMo/NF electrode exhibits outstanding HER performance, achieving 100 mA cm^−2^ at an overpotential of 127 mV and a high double-layer capacitance of 121.3 mF cm^−2^. Stability testing over 45 h at high current density confirmed excellent durability without noticeable degradation. These results underscore the benefits of combining the favorable electrochemical properties of NiMo alloys with the high surface area and conductivity of nickel foam, offering a promising strategy for the development of efficient, durable HER catalysts in clean energy applications.

## Data Availability

Data are contained within the article.
